# Case Report: Systemic Sclerosis After Covid-19 Infection

**DOI:** 10.3389/fimmu.2021.686699

**Published:** 2021-06-28

**Authors:** Serena Fineschi

**Affiliations:** ^1^ Department of Public Health and Caring Sciences, Unit of General Practice, Uppsala University, Uppsala, Sweden; ^2^ Östhammar Health Care Centre, Östhammar, Sweden

**Keywords:** COVID-19, scleroderma, systemic sclerosis, autoimmunity, long COVID

## Abstract

The coronavirus disease (COVID-19) is a respiratory tract infection caused by the new virus SARS-CoV-2. The acute phase of the infection may in certain individuals be followed by another longer phase of disease (long COVID) of unknown etiology probably associated in certain cases with autoimmune activation. It has been shown that COVID-19 can trigger autoantibody production and in genetically predisposed patients may cause the onset or exacerbation of autoimmune diseases. We are reporting a case of mild COVID-19 infection complicated by autoantibody production and cutaneous and gastrointestinal symptoms and subsequently diagnosed with systemic sclerosis (SSc). A 47-year-old man with no history of any autoimmune diseases and in good health became sick together with his family on the 12th of November with mild symptoms: tiredness, fever, cough, and sore throat. Oropharyngeal swab for SARS-CoV-2 tested positive. He was isolated at home and did not require hospitalization. Three weeks later he presented with clinical manifestation compatible with suspicion of SSc. He briefly presented with skin rush, periorbital edema and conjunctivitis, vomiting, dysphagia, burning sensation in the skin, above all in the fingertips and around the mouth, puffy fingers, Raynaud’s phenomenon, pain at the fingertip of the middle finger where a depressed area was noticed without a clear ulceration. ANA showed a strongly positive nucleolar pattern. Anti-PM/Scl 75 and PM/Scl 100 resulted positive. High-resolution computed tomography (HCRT) showed early stage of interstitial lung disease (ILD). The patient was diagnosed with SSc based on the persistence of autoantibodies and the clinical and radiological pictures according to the ACR/EULAR classification (scores: puffy finger, 2; ILD, 2; Raynaud’s phenomenon, 3; SSc related antibodies, 3; total 10). There are several cases described in the medical literature of possible new onset of SLE after COVID-19 infection. This is the first case that describes a possible new onset of SSc. Conclusion: SARS-CoV-2 may trigger systemic sclerosis.

## Introduction

Systemic sclerosis (SSc) is an autoimmune disease characterized by fibrosis in the skin and internal organs associated with vascular injury and immune system dysregulation (1). SSc is a rare condition that affects mostly young and middle-aged women, resulting in disproportionate morbidity and mortality. Vascular injury occurs early in the disease. Subsequent to vascular injury and immune disturbances, fibroblasts activate and produce collagen and extracellular matrix, resulting in organ dysfunction and tissue fibrosis. According to 2013 ACR/EULAR criteria, skin thickening of the fingers extending proximal to the metacarpophalangeal joints is sufficient for the patient to be classified as having SSc. If that is not present, seven additive items apply, with varying weights for each: skin thickening of the fingers, fingertip lesions, telangiectasia, abnormal nailfold capillaries, interstitial lung disease or pulmonary arterial hypertension, Raynaud’s phenomenon, and SSc-related autoantibodies. Sensitivity and specificity are high and also permit inclusion of patients whose disease is in the early stage. Patients having a total score of 9 or more are being classified as having definite systemic sclerosis (2). The most characteristic autoantibodies related to SSc are anti-centromere, anti-topoisomerase 1, and anti-RNA polymerase III. SSc can also overlap with other systemic autoimmune diseases, particularly with polymyositis. Anti-PM/Scl 75 and PM/Scl 100 are present in sera from patients with polymyositis (PM), systemic sclerosis, and PM/SSc overlap syndromes, and they may characterize distinct SSc subsets (3).

It is known that SSc can be triggered by viral illnesses and interferon signature (4). A viral infection can trigger autoimmunity through various mechanisms including molecular mimicry, activation of interferon-inducible genes, activation of apoptosis, epitope spreading and B-cell activation (5). In genetically predisposed patients, interferon activation can lead to the development of rapid autoimmune dysregulation resulting in autoimmune diseases (6, 7).

Coronavirus disease 19 (COVID-19) is an infection caused by a novel coronavirus SARS-CoV-2, which became a pandemic in 2020 after an initial outbreak in Wuhan, China. It is speculated that SARS-CoV-2 can disrupt self-tolerance and trigger autoimmune responses through cross-reactivity with host cells (8, 9). The debate is hot, since persistent or late symptoms after COVID-19 “long COVID “concern several million people. There are many similarities between COVID-19 disease and autoimmune diseases regarding clinical manifestations, immune response, and response to immunomodulatory treatment (10). Transient autoantibodies, in particular ANA, have been detected in COVID-19 patients (8, 11). Moreover, some patients have been reported to develop autoimmune diseases, such as Guillain–Barré syndrome or systemic lupus erythematosus, after COVID-19 infection (12–15). To my knowledge, no data exists on the onset of SSc after COVID-19 infection. A case report of deterioration of SSc in connection with COVID-19 infection with the occurrence of ANCA positive renal failure has been reported (16). However, the risk and prognosis of COVID-19 infection in patients with autoimmune diseases remain controversial. While most authors have not reported increased risk of severe COVID-19 infection in patients suffering from SSc (17), others have reported an increased risk in general concerning patients treated with immunosuppressive drugs, in particular rituximab (18).

We present a case of mild COVID-19 infection complicated with clinical cutaneous and gastrointestinal manifestation, autoantibody production, and radiological finding compatible with the diagnosis of systemic sclerosis (SSc).

### Case Description

**Figure 1:**
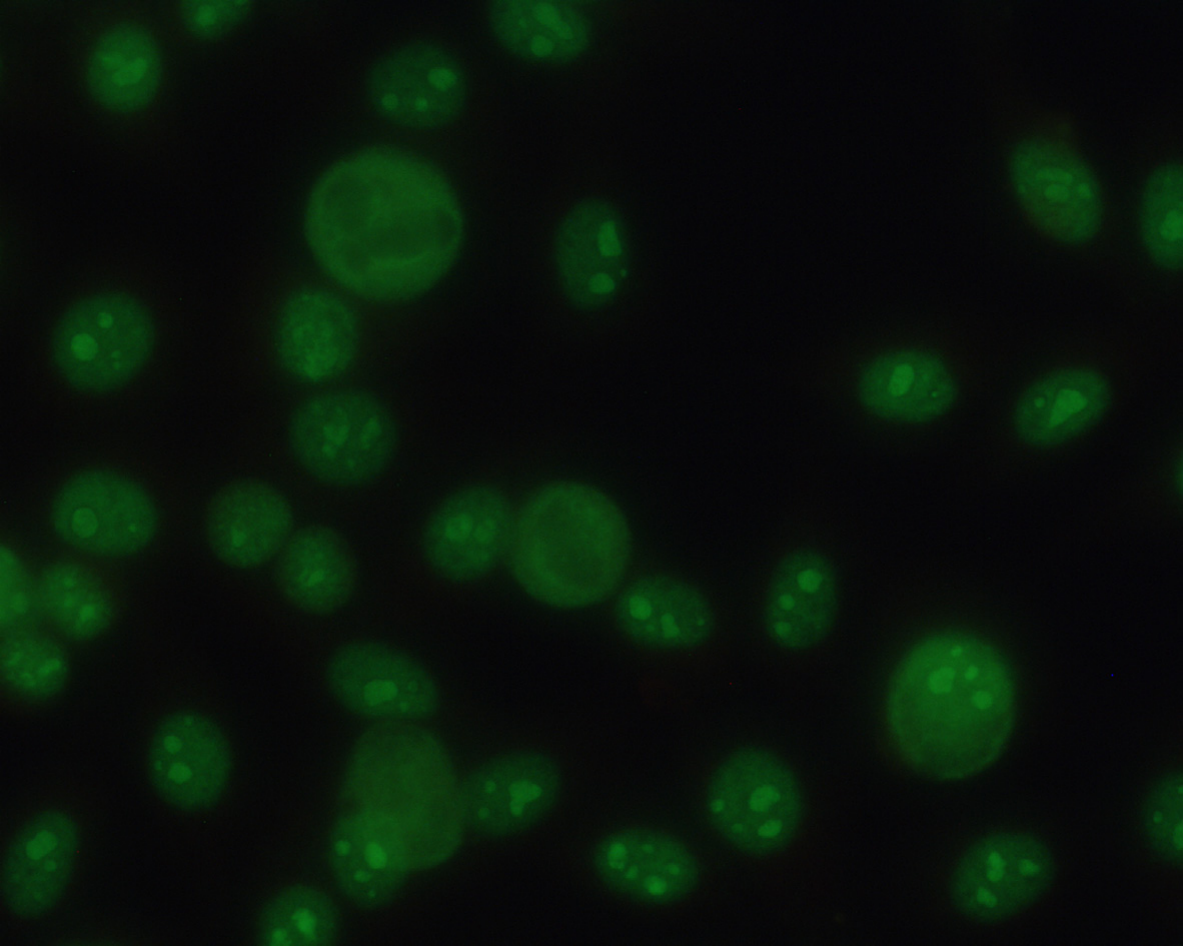
Indirect immunofluorescence assay on HEp-2 cells shows the following pattern: Homogeneous nucleolar/AC-8 (titer 1,600). According to ANA pattern classification Chan et al. (19).

**Figure 2:**
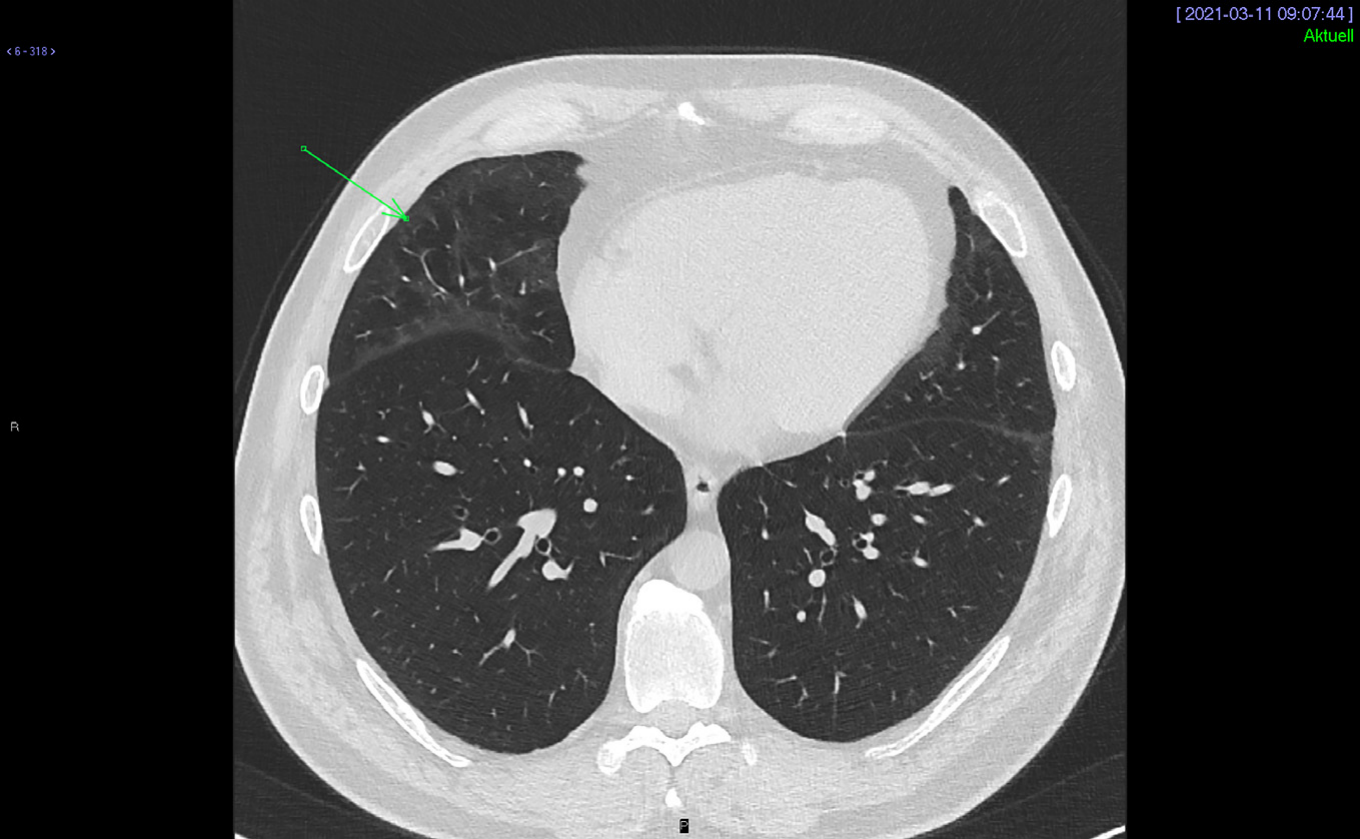
Peripheral subpleural ground-glass opacities compatible with early stage of interstitial lung disease (arrow) are present in the anterior and basal segments of the right middle lobe as well as along the oblique (major) fissure, and on the left side within the lingular segment.

A 47-year-old man with no history of any autoimmune diseases and in good health became sick together with his family on the 12th of November with mild symptoms of a flu-like disease: tiredness, fever, cough, and sore throat. Oropharyngeal swab for SARS-CoV-2 tested positive on the 17th of November. The patient was isolated at home and did not need hospitalization. Three weeks later his fever, cough, and sore throat had disappeared. However, he felt extremely tired and started to experience abdominal discomfort and redness of the skin. On the 1st of January he sought emergency care due to abdominal pain and vomiting, cutaneous generalized rash with diffuse erythema and burning skin sensation. He also had swelling of the eyelids and conjunctivitis. No abnormalities were detected on auscultation of the lungs and heart, and no lymphadenopathy or hepatosplenomegaly were found. The patient had no history of drug abuse; he was not taking any medication and did not smoke. ESR and CRP were normal as well as the complete blood count and serum creatinine. Chest radiography and oxygen saturation were normal, as well EKG, blood pressure, and heart rate. Patient was not hospitalized, and he returned home and started treatment with a proton pump inhibitor against acid reflux as well as antibiotic ointment against what we believed to be conjunctivitis and blepharitis. Patient only improved marginally, and two weeks later he sought care again. The cutaneous generalized rash had disappeared but was replaced by a heliotrope rash. He still had periorbital edema and conjunctivitis, burning sensation in the skin, above all in the fingertips and around the mouth. Patient complained of Raynaud’s phenomenon, swollen fingers and pain at the fingertip of the middle finger. He experienced a certain muscular discomfort and muscular weakness in the chest. Vomiting and abdominal pain had disappeared but the patient experienced dysphagia. At the physical examination, puffy fingers and a depressed area without clear ulceration at the fingertip of the middle finger were noticed. At this time, a large-scale analysis was taken. Complete blood count, ESR, complete metabolic panel, creatinine, muscle enzymes, thyroid function, rheumatoid factor, immunoglobulin, C-reactive protein, urinalysis, and complement levels were all normal. *H. pylori*, HCV, HBV, and HIV were negative. Immunofluorescent antinuclear antibody test using HEp-2 cells (ANA) showed strongly positive nucleolar pattern, and this raised suspicions of SSc (**Figure 1**). Specific autoantibodies were tested as the next step. Anti-PM/Scl 75 and PM/Scl 100 tested positive. Anti-Scl-70, anti-Jo 1, anti-RNA-polymerase III, and other autoantibodies tested negative (**Table 1**). The patient was diagnosed with SSc or scleroderma/polymyositis overlap (SSc-PM) and referred to rheumatologist. Echocardiography showed normal systolic artery pressure, and spirometry was normal. On the 11^th^ of March HCRT showed ground glass opacities with predominantly peripheral and subpleural distribution such as in the early stages of interstitial lung disease (ILD) (**Figure 2**). The rheumatologist confirmed the diagnosis of SSc (ACR/EULAR classification, score 10) and started a calcium channel blocker. After six months the patient still has puffy fingers and a depressed area at the fingertip of the middle finger that is pale, Raynaud’s phenomenon, some remaining heliotrope rash. He still has autoantibodies. A follow-up with HCRT, Schirmer Test, and labial salivary gland biopsy is scheduled. The treatment is currently calcium channel blocker, proton pump inhibitor, and tear substitution. The decision to start immunosuppressive therapy is being discussed and is dependent on the follow-up showing a progression of IDL.

**Table 1:** Autoantibodies.

Indirect immunofluorescence ANA	positive, 1: 1,600 nucleolar pattern
PM-Scl 75	positive
PM-Scl 100	positive
Sm	negative
U1RNP	negative
dsDNA	negative
Ribosomalt P	negative
Mi-2	negative
Ku	negative
SRP	negative
Jo-1	negative
PL-7	negative
PL-12	negative
EJ	negative
OJ	negative
TIF1-gamma	negative
MDA5	negative
NPX2	negative
SSA/Ro52	negative
SSA/Ro60	negative
SSB	negative
SAE1	negative
Scl-70	negative
CEMP-A	negative
CEMP-B	negative
RNA polymerase III, 11 kD	negative
RNA polymerase III, 155 kD	negative
Fibrillarin	negative
NOR90	negative
Th/To	negative
PDGFR	negative

## Discussion

This case report describes the onset of clinical symptom of SSc and autoantibodies after a mild COVID-19 infection in a previously healthy man.

The relationship between COVID-19 and the appearance of autoimmunity is complex and intricate. The diagnosis of SSc after COVID-19 can be problematic. The virus and the autoinflammatory response can be considered as two sides of the same coin. COVID-19 shares so many similarities with autoimmune diseases in clinical manifestations, immune responses, and pathogenic mechanisms that it may be difficult to distinguish which immune reactions/symptom/clinical manifestation are COVID-19 related and which are related to an autoimmune disease (20). There are some intriguing similarities between COVID-19 and SSc. Both diseases can be systemic, and high circulatory levels of IL-6, IL-10, and MCP-1 are found in both conditions (21, 22). COVID-19 infection can lead to endothelial damage, as well as SSc, and interstitial lung fibrosis is present in both.

Can a COVID-19-induced inflammation mimic SSc? Or is it a real SSc? The radiological finding of ground glass opacity may occur both in COVID-19 infection and in the early stage of non-specific interstitial pneumonia (NSIP) related to SSc (23). The patient had COVID-19 infection on the 12^th^ of November, and the HRCT was done on the 11^th^ of March. Since the time interval between onset of SSc and radiological chest CT scan is just three months it is reasonable to suppose that it can be an early stage of interstitial lung disease and not an advanced disease. The ANA positivity is described in high percentage of COVID-19 patients (8, 11). The nucleolar pattern has described often associated to interstitial pneumonia (8). Interestingly in these studies (8, 11) the autoantibody positivity correlates with poor prognosis and severe COVID-19 disease. It is unclear if the autoantibodies described in the literature are persistent or transitory, and it is unclear if they correlate with the symptoms and signs of autoimmune diseases or if they are just an epiphenomenon. In this case the autoantibodies are still present after six months and they correlate with specific symptoms and clinical findings of SSc. The specific symptom and clinical manifestation (*i.e.* puffy finger, Raynaud’s phenomenon, and dysphagia) correlate with a positive ANA, with the nucleolar pattern and a positive anti PM/Scl 75 and PM/Scl 100. Moreover, this patient was not critically ill due to COVID-19; he was not hospitalized, he did not experience any dyspnoea or desaturation, and he did not require any oxygen therapy. When the cough, sore throat, and fever disappeared, he experienced something else, something new. This is the second phase; the autoimmunity had replaced the initial viral symptoms.

It has been described that COVID-19 disease evolves in overlapping phases. The first is the viral phase that may be asymptomatic or mild. The second phase is the inflammatory phase where autoinflammation/autoimmunity can occur. The third phase is the hypercoagulability phase, and the fourth phase is characterized by organ damage (24). In this case the first and second phases are clearly distinguishable. The overlap was visible at the third week. The time of three weeks correlates well with the pathophysiology and antibody production. The temporary relationship between the two phases leads to the strong suspicious of causality between COVID-19 infection and SSc. Of course, there is always the possibility that the SSc was latent and the virus just exposed something that already existed. The patient, however, was in good health before.

Patient was diagnosed with SSc by a rheumatologist at the Uppsala University Hospital according to the ACR/EULAR classification (scores: puffy finger, 2; Raynaud’s phenomenon, 3; SSc-related antibodies, 3; interstitial lung disease, 2; total 10). It can be argued that PM/Scl 75 and PM/Scl 100 are not the classical SSc antibodies such as anti-centromere, anti-topoisomerase I, or anti-RNA polymerase III, but they are still quite common in SSc especially in the overlap form with polymyositis (3). Concerning the radiological finding of bilateral ground-glass opacities suggestive of NSIP, this is a typical radiological pattern in early SSc-associated ILD, but the subpleural distribution is unusual (25). Differential diagnosis with overlap syndrome may be taken in consideration.

Accurate follow-up is therefore necessary to confirm the SSc diagnosis and to differentiate it both from a transient autoinflammatory response to COVID-19 and from other autoimmune conditions that can overlap SSc. Despite the challenges in diagnostics, there is no doubt that COVID-19 infection has in this patient triggered an autoimmune process that is still active. At the six month follow-up, the clinical manifestation is still the same and the autoantibodies are still present. Long term follow-up is necessary in this mild and non-rapidly progressive case.

The mechanism of how SARS-CoV-2 triggers autoimmune diseases is under debate. Molecular mimicry due to the immune cross-reaction between epitopes and host antigens may be a possible explanation. Interferon production and cytokine activation can lead to disruption of immune tolerance in genetically predisposed subject. Defect of the function of dendritic cells that operate at the interface between innate and adaptive immunity is another possible mechanism (26). It is reasonable to suspect that COVID-19 has triggered SSc in this particular subject due to possible genetic predisposition. Candidate gene studies have identified critical immunoregulatory genes including *BANK1, FAM167A-BLK, IL23R, IRF5, STAT4, TBX21*, and *TNFSF4* as susceptibility genes for the development of SSc (27). It could be interesting to investigate if this patient has a specific genetic susceptibility. Genetic markers may help in the future to identify those subjects that run a higher risk of developing an autoimmune disease due to viral infections.

In conclusion, we want to emphasize the necessity to accurately investigate and to test autoantibodies in all the patients that present with unclear and new symptoms immediately after a COVID-19 infection. Autoimmune diseases may hide in the “long COVID” patient-group. It is possible that additional cases of SSc induced by COVID-19 will be reported in the near future.

## Data Availability Statement

The original contributions presented in the study are included in the article/supplementary material. Further inquiries can be directed to the corresponding author.

## Ethics Statement

Written informed consent was obtained from the patient for publication of this case report.

## Author Contributions

The author confirms being the sole contributor of this work and has approved it for publication.

## Conflict of Interest

The author declares that the research was conducted in the absence of any commercial or financial relationships that could be construed as a potential conflict of interest.
